# Activation of Transcription Factor EB Alleviates Tubular Epithelial Cell Injury via Restoring Lysosomal Homeostasis in Diabetic Nephropathy

**DOI:** 10.1155/2022/2812493

**Published:** 2022-01-12

**Authors:** Shujun Wang, Kaipeng Jing, Hongluan Wu, Xiaoyu Li, Chen Yang, Tingting Li, Haoxuan Tang, Ting Zou, Yao She, Hua-feng Liu

**Affiliations:** Key Laboratory of Prevention and Management of Chronic Kidney Disease of Zhanjiang City, Institute of Nephrology, Affiliated Hospital of Guangdong Medical University, Zhanjiang, Guangdong 524001, China

## Abstract

Disruption of lysosomal homeostasis contributes to the tubulopathy of diabetic nephropathy; however, its underlying mechanisms remain unclear. Herein, we report that decreased activity of transcription factor EB (TFEB) is responsible for the disturbed lysosome biogenesis and clearance in this pathological process. This was confirmed by the findings that insufficient lysosomal replenishment and damaged lysosomal clearance coincided with TFEB inactivation, which was mediated by mTOR hyperactivation in the renal tubular epithelial cells (TECs) of diabetic nephropathy. Furthermore, either TFEB overexpression or pharmacological activation of TFEB enhanced lysosomal clearance via promoting lysosomal biogenesis and protected TECs by reducing apoptosis *in vitro*. In addition, pharmacological activation of TFEB attenuated renal tubule injury, apoptosis, and inflammation in db/db mice. In conclusion, diabetes-induced mTOR activation represses TFEB function, thereby perturbing lysosomal homeostasis through impairing lysosomal biogenesis and clearance in TECs. Moreover, TFEB activation protects TECs from diabetic injuries via restoring lysosomal homeostasis.

## 1. Introduction

Diabetic nephropathy (DN) is the leading cause of end-stage renal disease and a threat to global health [1, 2]. Several novel agents targeting multifaceted pathogenic pathways of DN have been evaluated in recent clinical trials [3]. These agents include avosentan (a nonselective endothelin receptor antagonist), finerenone (a nonsteroidal mineralocorticoid receptor antagonist), baricitinib (a janus kinase 1/2 inhibitor), and selonsertib (an apoptosis signal-regulating kinase 1 inhibitor). However, among medications that are already widely used in patients with DN, only a few antidiabetic drugs, including renin angiotensin system (RAS) inhibitors, sodium-glucose cotransporter-2 (SGLT2) inhibitors, and glucagon-like peptide-1 receptor agonists, have demonstrated a potential renal benefits [4–6]. A more comprehensive understanding of DN pathogenesis may provide effective therapies in the future. Growing evidence suggests that tubular cells are not a victim but a neglected mediator in renal fibrosis in DN [7–10].

Advanced glycation end products (AGEs) are major pathogenic factor of diabetes mellitus and have been shown to induce epithelial-to-mesenchymal transition, hypertrophy, and apoptosis of tubular epithelial cells (TECs) [11–13]. Lysosome, as an integral part of the TEC molecular machinery, facilitates normal renal physiology [14]. Lysosomes are typically distinguished as primary and secondary lysosome [15]. Primary lysosomes originate from the Golgi apparatus and appear as small vesicles containing acid hydrolase. The fusion of Rab7 with lysosomal membrane proteins (LAMPs) converts primary lysosomes to secondary lysosomes, and the dissociation of Rab7 is necessary for lysosomal renewal [16]. Our previous research has indicated that AGEs impair lysosomal homeostasis, leading to lysosomal membrane permeabilization (LMP) and dysfunction, thus accelerating TEC injury by triggering apoptosis [17]. In addition, recent studies have revealed disturbed lysosomal membrane integrity and decreased lysosomal enzyme activities in TECs under DN conditions [18–20], suggesting that the disruption of lysosomal homeostasis participates in diabetic tubulopathy. However, the molecular mechanisms underlying this disruption remain largely unclear. Meanwhile, to sustain lysosomal stability, cells need not only to synthesize new lysosomal proteins and enzymes to compensate for the reduced lysosomal capacity [21], but also to efficiently remove damaged lysosomes via lysophagy in response to lysosomal stress under physiological conditions [22, 23]. Nevertheless, it is unclear whether there is any deficiency in this necessary lysosomal replenishment and/or clearance in TECs under diabetic conditions.

Transcription factor EB (TFEB) is considered as a master regulator of the coordinated lysosomal expression and regulation (CLEAR) gene network [24, 25], which is mainly regulated by the mammalian target of rapamycin (mTOR). Activated TFEB binds to the CLEAR network and drives the transcription of lysosome-related genes which encode proteins necessary for lysosomal biogenesis and function, to synthesize primary lysosomes and enhance lysosomal clearance [25]. Recently, the dysregulation of mTOR/TFEB signaling pathway has gained increasing recognition as a potential mechanism for development of several kidney diseases [26, 27], including DN [28]. However, it is not known whether pathogenic factors of DN in turn affect mTOR/TFEB-mediated lysosomal biogenesis and clearance, resulting in the impairment of lysosomal homeostasis in TECs and, if so, whether TFEB activation could maintain lysosomal homeostasis and thus alleviate TECs injuries under diabetic conditions. In the present study, we highlight the significance of TFEB in the lysosomal biogenesis and clearance in TECs of DN. Our results show that diabetes-induced mTOR activation contributes to TFEB dysfunction and therefore perturbs lysosomal homeostasis by impairing lysosomal biogenesis and clearance in TECs. Moreover, activation of TFEB attenuated TEC injuries by restoring lysosomal homeostasis in DN.

## 2. Materials and Methods

### 2.1. Ethics Statement and Patient Studies

The study protocol was approved by the Ethics Committee of the Affiliated Hospital of Guangdong Medical University (No. PJ2016048KY). The study of patients was conducted according to the Helsinki Declaration. Renal tissues from biopsies were obtained with informed written consent from the patients. Fresh renal samples were obtained from biopsy-confirmed DN patients (*n* = 15) also diagnosed with type 2 diabetes. Kidney specimens (*n* = 15) obtained from patients with mild urinary protein excretion or hematuria only and characterized as a minimal change disease in histology were used as controls. Clinical characteristics of patients are presented in Table 1, and average glycosylated hemoglobin (HbAlc) concentration was 5.99 ± 0.25 (mean ± SD) % for the DN group.

### 2.2. Cell Culture and Treatment

Human proximal tubular HK-2 cells (ATCC, CRL-2190_TM_) were cultured in Dulbecco's modified Eagle's medium (DMEM, Gibco, C11995500BT) supplemented with 10% fetal bovine serum (Gibco, 10270106). To investigate the effects of AGEs on TEC lysosomes, cultured HK-2 cells were exposed to 30, 50, and 100 *μ*g/ml nonglycated control bovine serum albumin (BSA) (Sigma, B2064) or AGE-BSA (EMD Millipore, 121800) for 12 h, respectively, prior to measurement. To simulate the activation of endogenous TFEB, HK-2 cells were pretreated with genistein (100 *μ*M; Sigma, G6649) for 12 h.

### 2.3. Lentivirus and Transduction

Human TFEB (NM_-_007162) and TFEB shRNA lentiviral particles were generated by Shanghai Genechem Co., Ltd. An empty vector was used as control. HK-2 cells were infected with the corresponding lentiviral supernatants for 12 h. Afterwards, the cells were cultured for another 72 h before being subjected to further analysis.

### 2.4. tfGalectin-3 Assay

As damaged lysosomes can be marked by galectin-3 in cells [29], the mRFP-GFP tandem fluorescent-tagged galactin-3 (tfGal3) plasmid (Addgene, #64149) was utilized to analyze the ability of cells to eliminate damaged lysosomes, as described previously [29]. Generally, GFP fluorescence is quenched under acidic conditions, whereas mRFP fluorescence is stable in the acidic luminal environment of lysosomes. Once LMP occurs, tfGal3 infiltrates into damaged lysosome lumen, binds the inner membrane, and illuminates both green and red fluorescence in an impaired acidic environment. Once damaged lysosomes are engulfed by autophagosomes and fused with intact lysosomes for degradation (a process termed lysophagy [30]), tfGal3 exhibits only a red signal due to the quenching of GFP fluorescence in an acidic environment. Thus, tfGal3 enables monitoring of the clearance of damaged lysosomes via lysophagy.

### 2.5. Mice

Animal experiment was approved by the Guangdong Medical University Animal Care and Use committee (No. GDY2016014). All mice were housed in the animal center of Guangdong Medical University in accordance with the Guide for the Care and Use of Laboratory Animals. The male C57BL/Ks db/db and age-matched nondiabetic (m/m) mice were purchased from the Biological Research Institute of Nanjing University and fed with a standard diet and had free access to water. At age of 6 weeks, the mice were divided into m/m (*n* = 6), db/db (*n* = 6), and db/db+genistein (*n* = 9) groups. Genistein was orally administered (dietary admix) once a day at dosage of 0.2 g/kg. Mice were anesthetized by intraperitoneal injection with an overdose of sodium pentobarbital and euthanized at 26 weeks. Kidney samples were collected for subsequent analyses.

### 2.6. Transmission Electron Microscopy (TEM)

TEM was used to measure the number and shape of lysosomes. Kidney tissue specimens or cells were fixed, embedded, and stained as previously described [31]. Ultrathin sections were examined using a Philips CM100 electron microscope (Eindhoven, The Netherlands). Primary lysosomes (electron-dense organelles filled with very tiny granules and usually spheres in three-dimensional shape) and secondary lysosomes (less electron-dense and large heterogeneous more or less spherical organelles) [32] were identified by two individuals, blinded to the study.

### 2.7. RNA Extraction and Quantitative Real-Time PCR

Total RNA was extracted using TRIzol reagent (TaKaRa, #9109), and cDNA was synthesized using a reverse transcription- (RT-) quantitative kit (TaKaRa, RR047A) according to the manufacturer's protocols. The mRNA levels of genes of interest were quantified by PCR using primer sequences listed in supplementary material (Table S1).

### 2.8. Western Blot

Total cell lysates were obtained as described previously [33]; the nuclear and cytosolic fractions were obtained using a nuclear and cytoplasmic protein extraction reagent (Sigma, 9002-93-1). Western blot analysis was performed as described previously [33]. Primary antibodies included are the following: TFEB (BETHYL, A303-672A), p-TFEB (Merck Millipore, ABE1971), LC3B (Abcam, Ab51520), p62/SQSTM1 (Abcam, Ab25631), p-mTOR (Cell Signaling, 5536), PARP (Cell Signaling, 9532), BCL-2 (Abcam, Ab196495), Bax (Cell Signaling, 2772), IL-1*β* (R&D system, AF401), caspase 1 (Abcam, ab179515), caspase 3 (Cell Signaling, 9664), tubulin (Santa Cruz, sc-32293), *β*-actin (Abcam, ab8227), and nuclear matrix protein p84 (Gene Tex, GTX70220). All primary antibodies were diluted to 1: 1000. Blots were imaged with the C500 Imaging System (Azure Biosystems) in automatic exposure mode. The densities of protein signals were quantified using Image J (NIH), and all the blots were normalized to the loading control.

### 2.9. Immunofluorescence Staining

The kidney samples or cells were fixed, embedded, and stained as previously described [34]^27^. The primary antibodies against TFEB (BETHYL, A303-672A), LC3B (Abcam, Ab51520), p62/SQSTM1 (Abcam, Ab56416), lysosomal-associated membrane protein 2 (LAMP2; Abcam, Ab25631), Rab7 (Abcam, Ab137026), and mTOR (Cell Signaling, 2983) AGE-BSA (Abcam, ab23722) were used. All primary antibodies were diluted to 1: 100. Images were taken by a TCS SP5II confocal microscope (Leica Microsystems, Wetzlar, Germany). The integral optical density (IOD) for at least five fields of each slide was evaluated by Image-Pro Plus image analysis software version 6.0.

### 2.10. Ovalbumin Dequenching Assay

The proteolytic degradation of lysosomes was assessed using DQ-ovalbumin (Invitrogen, D12053), which forms green fluorescent puncta once it is degraded within lysosomes, according to the manufacturer's protocols. Briefly, after treatments, HK-2 cells were incubated with DQ-ovalbumin (10 *μ*g/ml) at 37°C for 2 h. Then, the cells were washed with PBS and fixed with 4% paraformaldehyde. Alternatively, the cells were trypsinized and subjected by flow cytometry to measure the fluorescent intensity value of DQ-ovalbumin using a FACSCanto II platform (BD Bioscience, USA).

### 2.11. FITC Annexin V/PI Assays for HK-2 Cell Apoptosis

Cells were stained with an Annexin V/PI Apoptosis Detection Kit (Dojindo, AD10) according to the manufacturer's protocols and then analyzed using the FACSCanto II platform mentioned above.

### 2.12. Renal Function

Total urinary albumin excretion was measured using a hydrochlorophenol red assay according to the manufacturer's instructions (LEADMAD, CS9480), and urinary creatinine was measured by a picric acid method according to the manufacturer's instructions (Roche Diagnostics GmbH, 06407137190). Urinary albumin excretion was calculated as total urinary albumin/creatinine ratio (ACR). Serum creatinine levels were detected by a creatinine assay kit (Nanjing Jiancheng Bioengineering Institute, C011-2-1).

### 2.13. Immunohistochemistry

Immunohistochemical staining for kidney injury molecule-1 (Kim-1) was performed as described previously [35]. Primary antibodies against Kim-1 (R&D, AF1817) were used and diluted to 1 : 200 for staining. Sections were counterstained with hematoxylin to stain the nuclei. The area of Kim-1 positive tubules was estimated and counted in at least 15 randomly selected high-power fields (×400). The Image J software was utilized to quantify the positive signals.

### 2.14. Statistical Analysis

All statistical tests were performed with SPSS statistical software version 16.0. Results are expressed as the means ± SEM of at least three independent experiments. If the data were normally distributed, independent-sample *t*-test was used to analyze significance between two groups. One-way ANOVA followed by Tukey multiple comparison tests was used to analyze the significance between multiple groups. The Wilcoxon rank sum test was used for the counting data. Statistically significant was set at *P* < 0.05.

## 3. Results

### 3.1. Deficiency in Lysosomal Replenishment and Clearance in TECs under Diabetic Conditions

**Figure 1:**
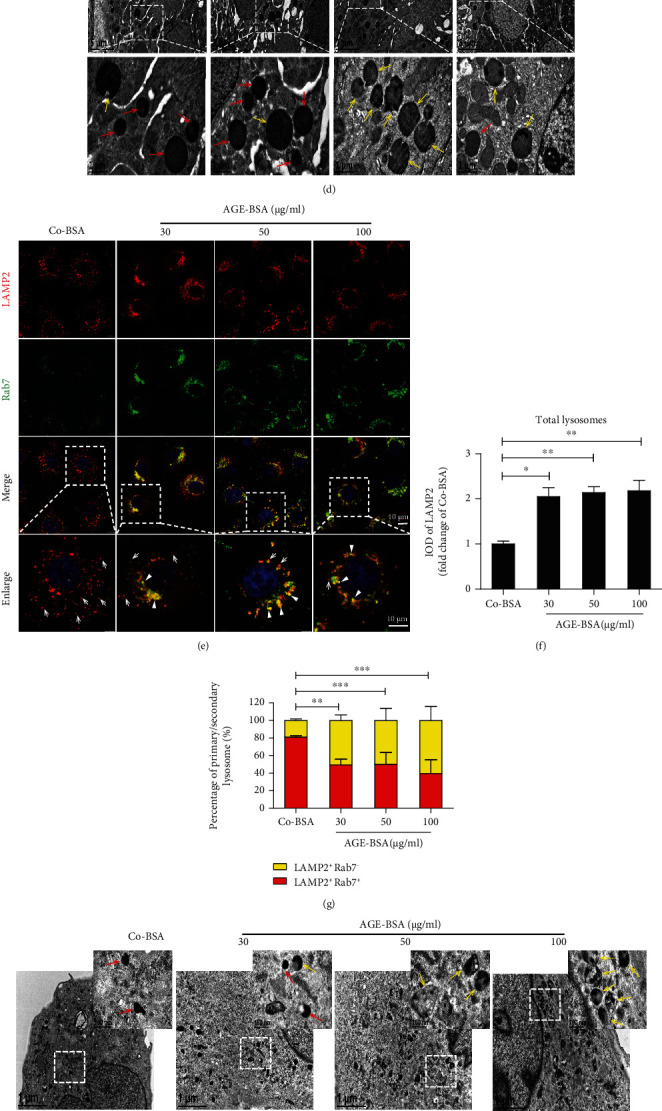
Deficiency in lysosomal replenishment and clearance in TECs under diabetic conditions. (a) Immunofluorescence staining for LAMP2 and Rab7 to detect primary or secondary lysosomes in renal cortex of kidney biopsy specimens from DN patients and controls. LAMP2-positive puncta in the top panel represent total lysosomes, and LAMP2 and Rab7 double-positive puncta represent secondary lysosomes (arrowheads), while LAMP2-positive puncta alone in the merged image represent primary lysosomes (arrows). DAPI was used to stain the nuclei. Scale bar, 10 *μ*m. (b, c) Quantitative data of the fluorescence integral optical density (IOD) for total lysosomes and the percentage of primary and secondary lysosomes in renal cortex. Data are presented as mean ± SEM (*n* = 15). (d) TEM images of kidney specimens from patients with DN or controls. Boxed areas are magnified and shown in the bottom panel. Primary lysosomes: red arrows and secondary lysosomes: yellow arrows. (e) Immunofluorescence staining of LAMP2 and Rab7 in HK-2 cells incubated with increasing concentrations (0, 30, 50, and 100 *μ*g/ml) of AGE-BSA for 12 h. DAPI was used to stain the nuclei. Scale bar, 10 *μ*m. (f) Fluorescence integral optical density (IOD) for total lysosomes and (g) percentage of primary (arrows) and secondary lysosomes (arrowheads). (h) Representative TEM images of the lysosome changes in HK-2 cells incubated with indicated concentrations of AGE-BSA for 12 h. Boxed areas are magnified and shown in the upper right panel. Primary lysosomes: red arrows and secondary lysosomes: yellow arrows. (i, j) Representative histogram obtained from DQ-ovalbumin staining followed by flow cytometric analysis in HK-2 cells treated with indicated concentrations of AGE-BSA for 12 h. Bars represent the mean ± SEM for at least three independent experiments. ^∗^*P* < 0.05, ^∗∗^*P* < 0.01, and ^∗∗∗^*P* < 0.001.

To determine whether lysosomal replenishment and/or clearance is involved in DN, we first examined changes in the number of primary and secondary lysosomes in TECs by conducting double immunofluorescence staining using LAMP2 and Rab7 (Figures 1(a)–1(c)). We found that there were a large number of lysosomes positive for LAMP2 (total lysosomes) within TECs of patients with DN, and substantially, almost all these lysosomes were secondary lysosomes as they were also positive for Rab7. Meanwhile, the percentage of primary lysosomes identified by the expression of LAMP2 alone was significantly decreased. These changes were further confirmed by TEM. As shown in Figure 1(d), the number of primary lysosomes, which are electron-dense vesicles homogenously filled with tiny granules, decreased, while the number of secondary lysosomes, which are irregular in appearance with variable electron density, increased remarkably. The observed lack of primary lysosomes implies the insufficiency of lysosomal replenishment, and the increased number of secondary lysosomes suggests lysosomal clearance might be impeded.

To confirm this, we further treated HK-2 cells with AGE-BSA to mimic DN conditions, and the changes in the number of primary and secondary lysosomes were evaluated by dual immunofluorescence staining for LAMP2 and Rab7. We found the percentage of primary lysosomes (puncta positive for LAMP2 alone) was indeed reduced with a concurrent increase in the percentage of secondary lysosomes (vesicles positive for both LAMP2 and Rab7) (Figures 1(e)–1(g)). Very similar results were also obtained by TEM (Figure 1(h)). Likewise, as adequate functional lysosome is important for the maintenance of lysosomal degradation capacity [36], we next evaluated the efficiency of lysosome-mediated proteolytic degradation of DQ-ovalbumin. As expected, AGE-BSA treatment significantly impaired DQ-ovalbumin degradation in a dose-dependent manner (Figures 1(i) and 1(j)). Together, these results indicate that lysosome replenishment and clearance are impaired under diabetic conditions.

Lysosomal clearance is essential for the degradation of substrates delivered via autophagy [37]. To explore whether deficient lysosomal clearance would impair autophagic degradation, the expression patterns of LC3 II (a key marker of autophagy) and p62 (a known autophagy substrate) were examined. Immunofluorescence assays revealed a significant accumulation of both LC3 II and p62 positive vesicles in renal tubule cells from patients with DN, as well as in db/db mice and AGE-treated HK-2 cells (Fig. S1 a, b, d). Western blot analysis also revealed increased protein levels of LC3 II and p62 in renal tissues of db/db mice and in AGE-treated HK-2 cells (Fig. S1 c, e). These results indicate that lysosomal clearance was indeed deficient in diabetic TECs.

### 3.2. Decreased TFEB Expression and Activity in Diabetic TECs

**Figure 2:**
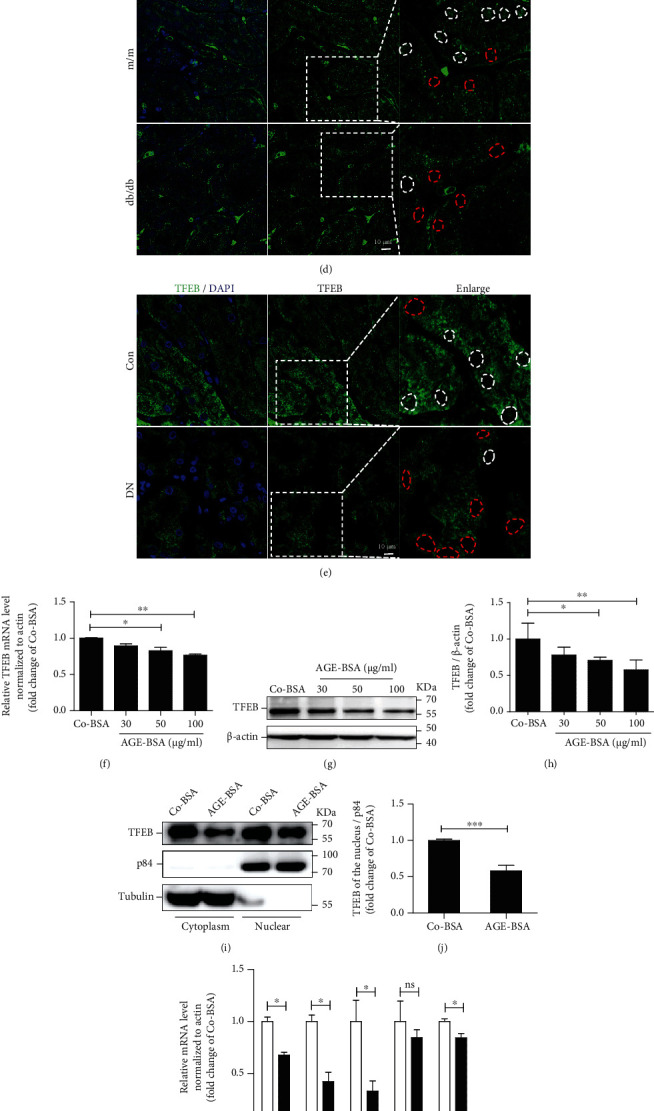
Decreased TFEB expression and activity in TECs under diabetic conditions. (a) Real-time PCR analyses of TFEB mRNA expression in kidney from db/db or m/m mice. (b, c) Western blot assay and quantitative analysis of TFEB protein levels in kidneys. (d, e) Immunofluorescence staining for TFEB in renal cortex of mice and patients. Scale bar, 10 *μ*m. The white dotted lines represent TFEB nuclear expression, and the red dotted lines represent decreased TFEB nuclear expression. Boxed areas are magnified and shown in the right panel. (f) Quantification of TFEB levels in HK-2 cells after exposure to increasing concentrations (0, 30, 50, and 100 *μ*g/ml) of AGE-BSA for 12 h by real-time PCR and (g, h) western blot. (i, j) Subcellular distribution of TFEB in HK-2 cells treated with Co-BSA or AGE-BSA (50 *μ*g/ml) for 12 h. (k) Expression levels of TFEB target genes (ATPV1A, VPS11, CLCN7, GBA, and ATPV1B) analyzed by real-time PCR in HK-2 cells treated with Co-BSA or AGE-BSA (50 *μ*g/ml) for 12 h. Bars represent the mean ± SEM for at least three independent experiments. ^∗^*P* < 0.05 and ^∗∗^*P* < 0.01.

We investigated whether TFEB expression and/or activity is involved in the deficient lysosomal replenishment and clearance observed in diabetic TECs. We found the mRNA and protein levels of TFEB were lower in kidneys isolated from db/db mice, compared with that from m/m mice (Figures 2(a)–2(c)). Furthermore, immunofluorescence assays revealed a diminished expression of TFEB in nucleus of TECs from db/db mice (Figure 2(d)) and patients with DN (Figure 2(e)). Consistent with these findings *in vivo*, concentration-dependent decreases in TFEB mRNA and protein levels were found in HK-2 cells treated with AGE-BSA, as determined by real-time PCR and western blot assays, respectively (Figures 2(f)–2(h)). In parallel, a decreased level of nuclear TFEB protein was found in HK-2 cells after AGE-BSA stimulation (Figures 2(i) and 2(j)). We also found that AGE-BSA downregulated the mRNA levels of TFEB target genes (Figure 2(k)), which are known to mediate lysosomal substrate degradation (*Vps11*) and lysosomal acidification (*CLCN7*, *ATP6V1A*, and *ATP6V1B*). These data clearly indicate that TFEB was inactivated in renal TECs under diabetic conditions.

### 3.3. Activation and Augmented Lysosomal Translocation of mTOR in Diabetic TECs

**Figure 3:**
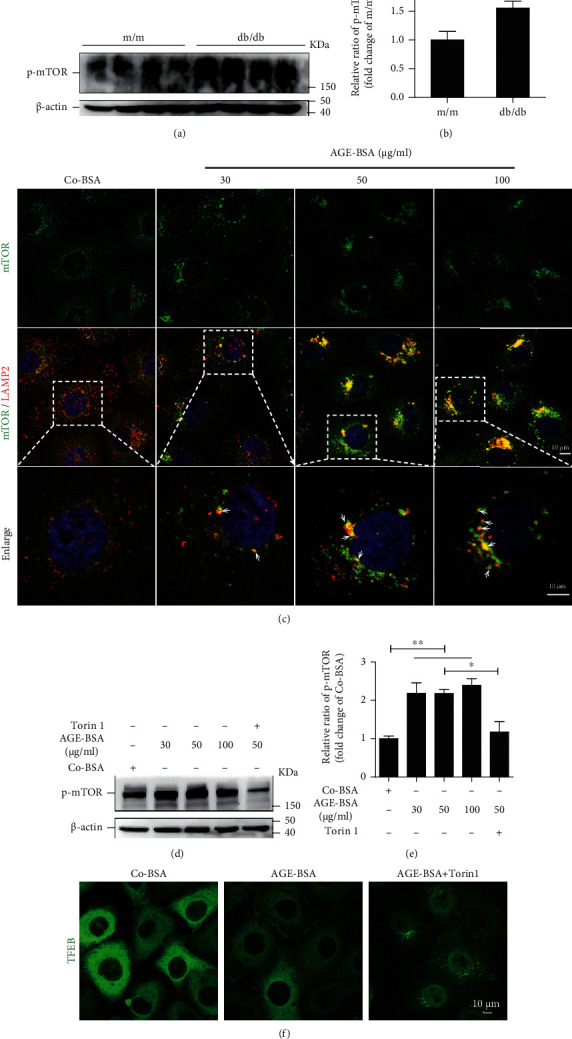
mTOR is activated in TECs under diabetic condition. (a, b) Western blot assay and quantification of p-mTOR level in renal tissues from db/db and m/m mice. Data are presented as mean ± SEM. (c) Immunofluorescence staining of mTOR and LAMP2 in HK-2 cells after exposure to increasing concentrations of AGE-BSA for 12 h. Representative of images is shown. Arrows denote colocalization of mTOR with LAMP2. DAPI was used to stain the nuclei. Scale bar, 10 *μ*m. Boxed areas are magnified and shown in the bottom panel. (d, e) Western blot assay and quantification of p-mTOR level in HK-2 cells after exposure to increasing concentrations of AGE-BSA alone or AGE-BSA (50 *μ*g/ml) plus Torin1 (1.5 *μ*M) for 12 h. Bars represent the mean ± SEM for at least three independent experiments. (f) Immunofluorescence staining of TFEB in HK-2 cells, which were treated with Co-BSA and AGE-BSA (50 *μ*g/ml) and AGE-BSA (50 *μ*g/ml) plus Torin1 (1.5 *μ*M) for 12 h, respectively. ^∗^*P* < 0.05 and ^∗∗^*P* < 0.01.

The level of phosphorylated mTOR, which represents the active status of mTOR, was increased in kidneys of db/db mice (Figures 3(a) and 3(b)). We also found that AGEs led to a punctate pattern of mTOR which colocalized with LAMP2 in HK-2 cells (Figure 3(c)), suggesting that the lysosomal translocation of mTOR is increased in diabetic TECs. Furthermore, AGEs markedly elevated mTOR activity, as evidenced by an increased level of phosphorylated mTOR, which was significantly reversed by an mTOR inhibitor, Torin1 (Figures 3(d) and 3(e)). Importantly, Torin1 also promoted TFEB nuclear translocation in AGE-treated HK-2 cells (Figure 3(f)). Together, these results suggest that mTOR was hyperactivated and acted as a negative mediator of TFEB in diabetic TECs.

### 3.4. TFEB Overexpression Enhanced Lysosomal Clearance via Promoting Lysosomal Biogenesis and Protected TECs from the Diabetes-Induced Injury

**Figure 4:**
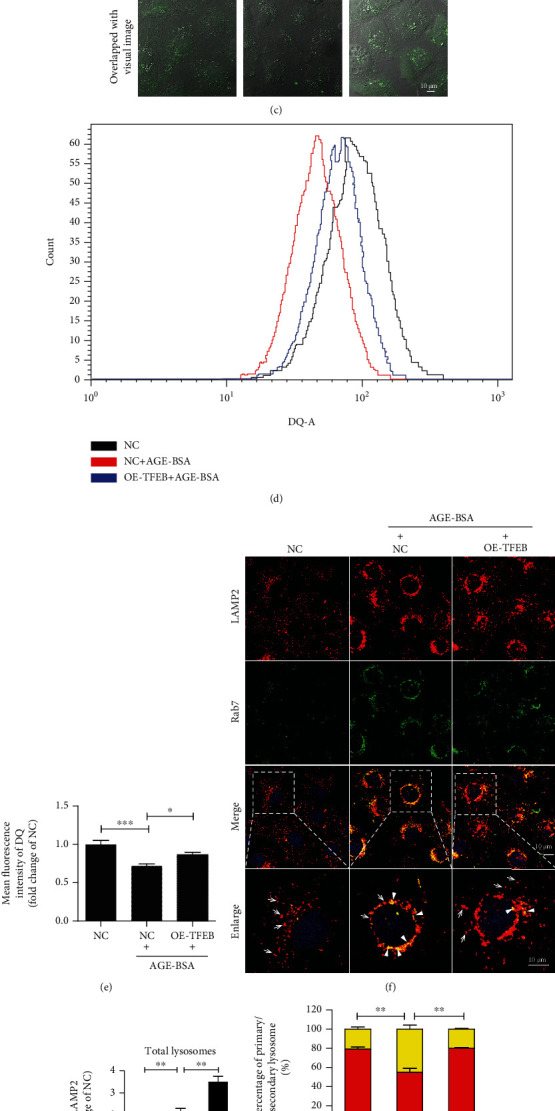
Effect of TFEB overexpression on lysosomal clearance, lysosomal biogenesis, and AGE-induced apoptosis in TECs. (a) Fluorescent microscopy analysis of lysosomal damage and clearance in RFP-GFP-Galectin-3-transfected HK-2 cells. After being infected by lentivirus carrying with or without TFEB, HK-2 cells were further transiently transfected with RFP-GFP-Galectin-3 plasmids and then exposed to AGE-BSA (50 *μ*g/ml) for 12 h. The yellow dots represent damaged lysosomes (arrowheads), while the red puncta indicate functional lysosomes with lysophagic degradation of damaged lysosomes (arrows). DAPI was used to stain the nuclei. Boxed areas are magnified and shown in the bottom panel. (b) Quantitative data for yellow dots or free red dots per cell. (c) Immunofluorescence staining and (d, e) flow cytometry analysis of DQ-ovalbumin in AGE-BSA (50 *μ*g/ml) treated HK-2 cells with or without TFEB overexpression. (f) Immunofluorescence staining LAMP2 and Rab7 to detect primary lysosomes (arrows) or secondary lysosomes (arrowheads) in TFEB overexpressing HK-2 cells treated with or without AGE-BSA (50 *μ*g/ml) for 12 h. DAPI was used to stain the nuclei. Boxed areas are magnified and shown in the bottom panel. (g, h) Fluorescence integral optical density (IOD) for total lysosomes and the percentage of primary and secondary lysosomes. (i, j) Flow cytometry analysis and quantitative data for apoptosis in HK-2 cells. (k, l) Immunoblotting analysis and quantitative data for PARP protein in HK-2 cells. Scale bar, 10 *μ*m. NC: negative control; OE-TFEB: overexpressing TFEB. ^∗^*P* < 0.05, ^∗∗^*P* < 0.01, and ^∗∗∗^*P* < 0.001.

The tfGal3 plasmid was employed to investigate whether TFEB overexpression enhances the clearance of damaged lysosomes. As shown in Fig. S2, TFEB overexpression by lentivirus infection effectively increased the mRNA and protein levels of TFEB. These cells were then subsequently transfected with the tfGal3 plasmids and treated with or without AGE-BSA (Figures 4(a) and 4(b)). Fluorescence microscopy demonstrated that tfGal3 was distributed diffusely into the cytoplasm of negative control HK-2 cells, while AGE-BSA exposure led to a significant formation of yellow puncta (mRFP^+^GFP^+^), suggesting severe damage to lysosomes. Intriguingly, TFEB overexpression inhibited the formation of yellow dots, induced by AGE-BSA, and elevated the number of red dots, implying that TFEB overexpression enhances the clearance of damaged lysosomes in TECs. Given that lysosomal degradation is essential for the lysosomal clearance, we hypothesized that TFEB overexpression would also rescue the impaired lysosomal degradation capacity under diabetic conditions. We found that AGE-BSA treatment significantly impaired DQ-ovalbumin degradation as evidenced by a decrease in green fluorescent signal, which could be partially rescued by TFEB overexpression (Figures 4(c)–4(e)).

We then tested whether TFEB overexpression could restore the lysosomal system by promoting lysosome biogenesis. As shown in Figures 4(f)–4(h), HK-2 cells overexpressing TFEB exhibited a significant reduction in the number of secondary lysosomes with an increased number of primary lysosomes after AGE-BSA stimulation, as evidenced by the decreased number of yellow puncta (positive for LAMP2 and Rab7) and a concurrent increase in LAMP2-only positive vesicles. These data demonstrate that TFEB overexpression promoted lysosomal biogenesis.

In addition, TFEB overexpression reduced the AGE-induced apoptosis in HK-2 cells, as evidenced by reductions in the Annexin V+/PI+ portion (Figures 4(i) and 4(j)) and decreased levels of PARP cleavage (Figures 4(k) and 4(i)), suggesting that TFEB overexpression protected TECs from AGE-induced injury.

### 3.5. Effect of TFEB Activation on Lysosomal Biogenesis and Lysosomal Clearance In Vitro and in db/db Mice

**Figure 5:**
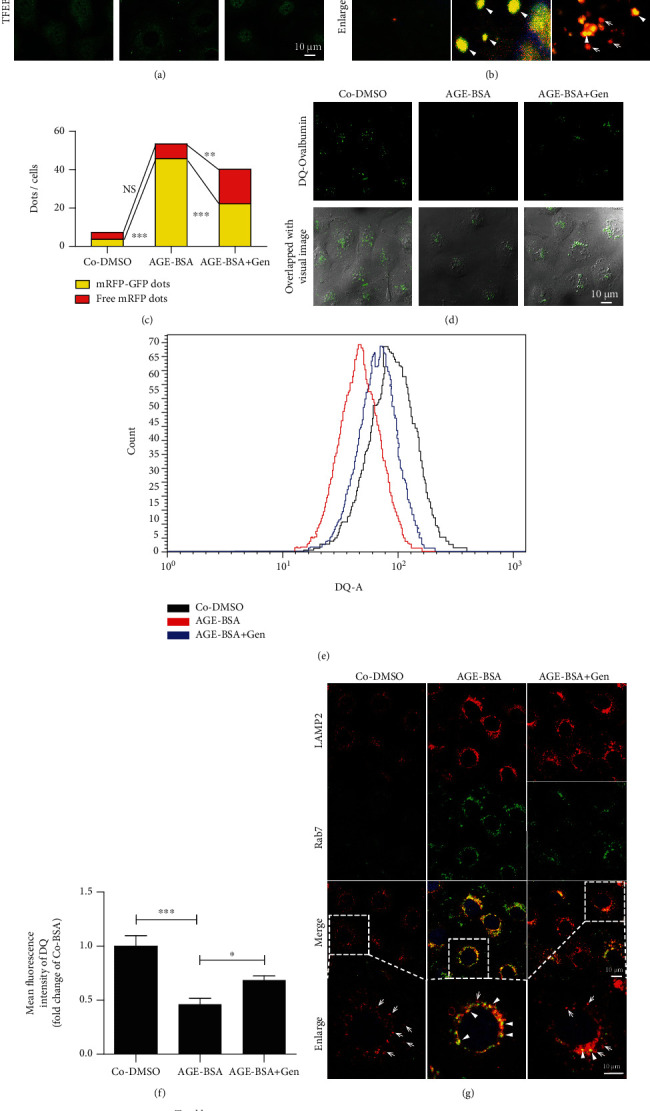
Nuclear translocation of TFEB trigger by genistein promotes the lysosomal biogenesis and increases lysosomal clearance in diabetic TECs in vitro. (a) Distribution of TFEB in HK-2 cells pretreated with or without 100 *μ*M genistein followed by Co-DMSO or AGE-BSA (50 *μ*g/ml) exposure for 12 h. (b) Lysosome damage and clearance in HK-2 cells. Cells were transfected with RFP-GFP-Galectin-3, subsequently treated with or without 100 *μ*M genistein, and then exposed to Co-DMSO or AGE-BSA (50 *μ*g/ml) for 12 h. The yellow dots represent damaged lysosomes (arrowheads), while the red puncta indicate functional lysosomes with lysophagic degradation of damaged lysosomes (arrows). DAPI was used to stain the nuclei. (c) Quantitative data for yellow dots or free red dots per cell. (d–f) Immunofluorescence staining and flow cytometry analysis of DQ-ovalbumin in HK-2 cells with indicated treatments. (g) Immunofluorescence staining of LAMP2 and Rab7 to detect primary lysosomes (arrows) or secondary lysosomes (arrowheads) in HK-2 cells. (h, i) Fluorescence integral optical density (IOD) for total lysosomes and percentage of primary and secondary lysosomes per cell. DAPI was used to stain the nuclei. Scale bar, 10 *μ*m. Gen: genistein. ^∗^*P* < 0.05, ^∗∗^*P* < 0.01, and ^∗∗∗^*P* < 0.001.

**Figure 6:**
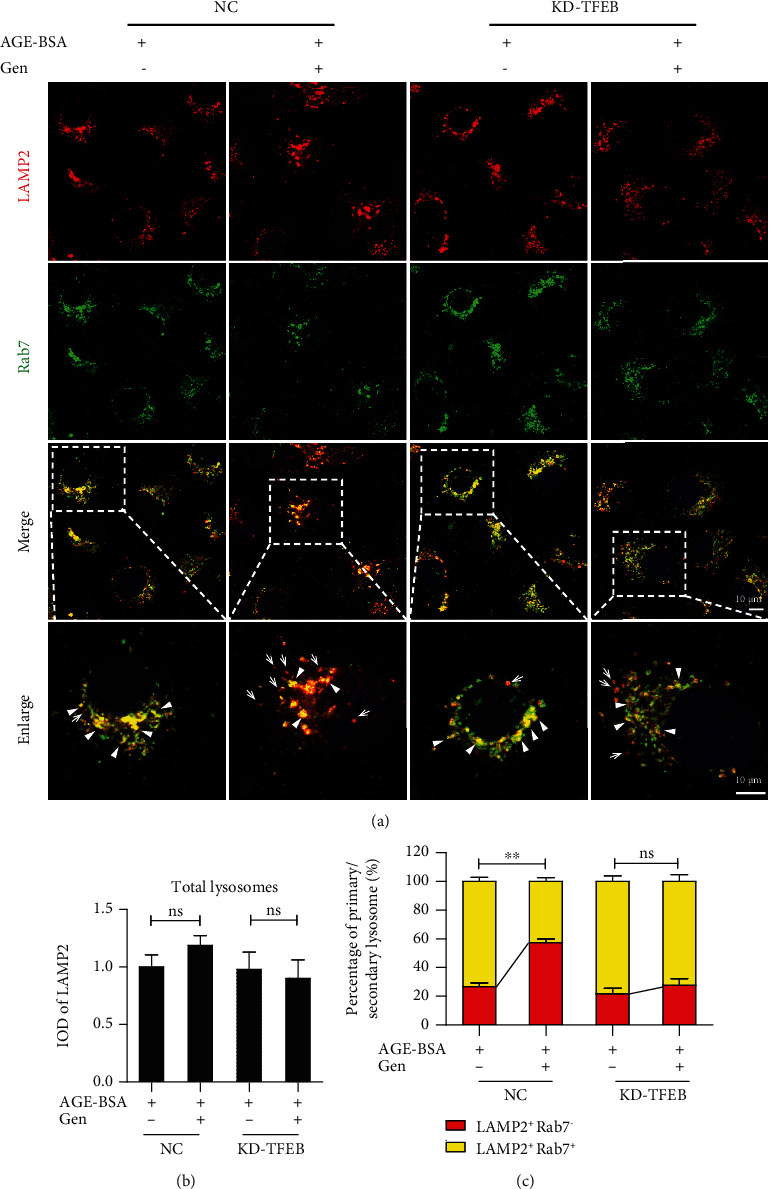
Knockdown of TFEB abolishes the genistein-induced lysosomal biogenesis in HK-2 cells. HK-2 cells with or without TFEB knockdown were exposed to AGE-BSA (50 *μ*g/ml) in the presence or absence of genistein (100 *μ*M) for 12 h. (a) Immunofluorescence staining of LAMP2 and Rab7 to detect primary lysosomes (arrows) or secondary lysosomes (arrowheads) in HK-2 cells. (b, c) Fluorescence integral optical density (IOD) for total lysosomes and the percentage of primary and secondary lysosomes per cells. DAPI was used to stain the nuclei. Scale bar, 10 *μ*m. Gen: genistein; NC: negative control; KD-TFEB: TFEB knockdown. ^∗∗^*P* < 0.01.

We then investigated the impact of TFEB activation on lysosomal system in diabetic TECs using a known TFEB activator, genistein [38]. As shown in Figure 5(a), genistein markedly increased TFEB nuclear translocation in AGE-treated HK-2 cells. We then evaluated whether TFEB activation by genistein treatment could eliminate the accumulation of damaged lysosomes in diabetic TECs. In tfGal3-transfected HK-2 cells, the number of yellow puncta significantly increased after AGE stimulation (Figures 5(b) and 5(c)), whereas genistein pretreatment could decrease the amount of yellow puncta induced by AGEs and augmented the number of red puncta, suggesting that clearance of damaged lysosomes was increased in diabetic TECs. In addition, immunofluorescent staining and flow cytometric analysis revealed that genistein treatment restored the AGE-induced impairment of DQ-ovalbumin degradation (Figures 5(d)–5(f)). Furthermore, genistein treatment promoted lysosomal biogenesis, as evidenced by a significant increase in the number of primary lysosomes in HK-2 cells (Figures 5(g)–5(i)). Conversely, the positive effect of genistein on lysosomal biogenesis was remarkably abrogated in TFEB-knockdown HK-2 cells treated with AGEs (Figure 6).

**Figure 7:**
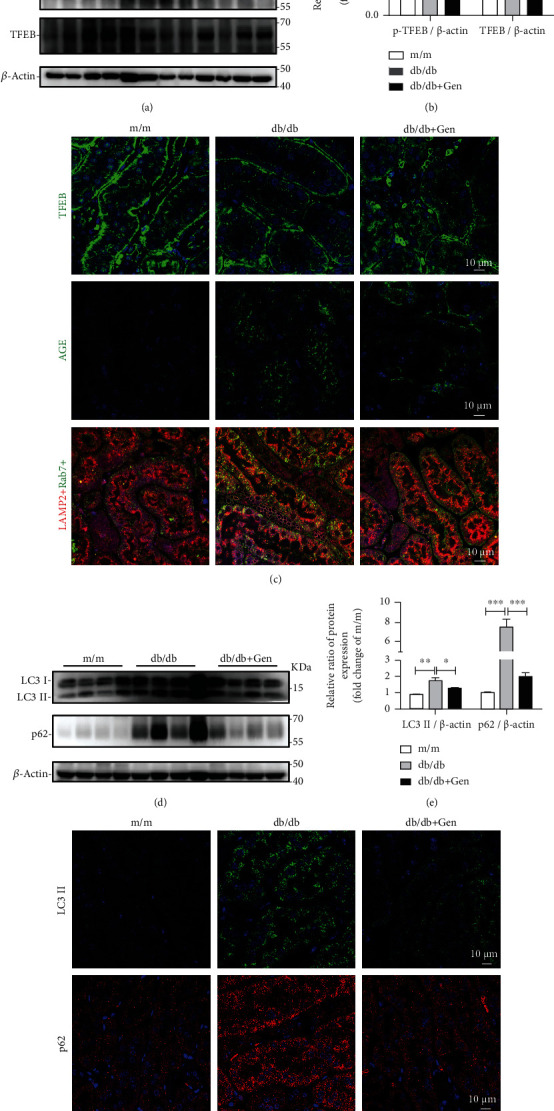
TFEB activation by genistein promotes lysosomal biogenesis, reduces the expression of AGE, and alleviates autophagic blockage in db/db mice. (a, b) Western blot assay and quantification of p-TFEB and TFEB levels in renal tissues from m/m and db/db mice treated with or without genistein. (c) Representative images of immunofluorescence staining for TFEB, AGE, LAMP2, and Rab7 in renal cortex from mice. Scale bar, 10 *μ*m. (d, e) Western blot assay and quantification of LC3 and p62 levels in murine renal tissues. (f) Representative images of immunofluorescence staining with anti-LC3 and p62 antibodies in renal cortex from mice. Gen: genistein. ^∗^*P* < 0.05, ^∗∗^*P* < 0.01, and ^∗∗∗^*P* < 0.001.

We found genistein treatment decreased the phosphorylation of TFEB in db/db mouse kidneys (Figures 7(a) and 7(b)). Concurrently, immunofluorescence assays revealed elevation of TFEB nuclear translocation, reduced accumulation of AGE, and increased lysosomal biogenesis, as identified by the number of LAMP2-only positive dots was increased in db/db renal TECs, which was associated with an increasing TFEB nuclear translocation (Figure 7(c)). Along with this, the results obtained from immunofluorescence staining and western blot analysis showed genistein treatment notably reduced the accumulation of autophagic vacuoles, as evidenced by a decreased number of LC3 II and p62-positive puncta in renal TECs, as well as the reduced levels of these proteins in kidneys of db/db mice (Figures 7(d)–7(f)). Overall, these data reveal that TFEB activation with genistein promoted autophagy degradation by triggering lysosomal biogenesis and increasing the clearance of injured lysosomes in diabetic TECs.

### 3.6. Effect of TFEB Activation on Renal Injury, Apoptosis, and Inflammation in db/db Mice

**Figure 8:**
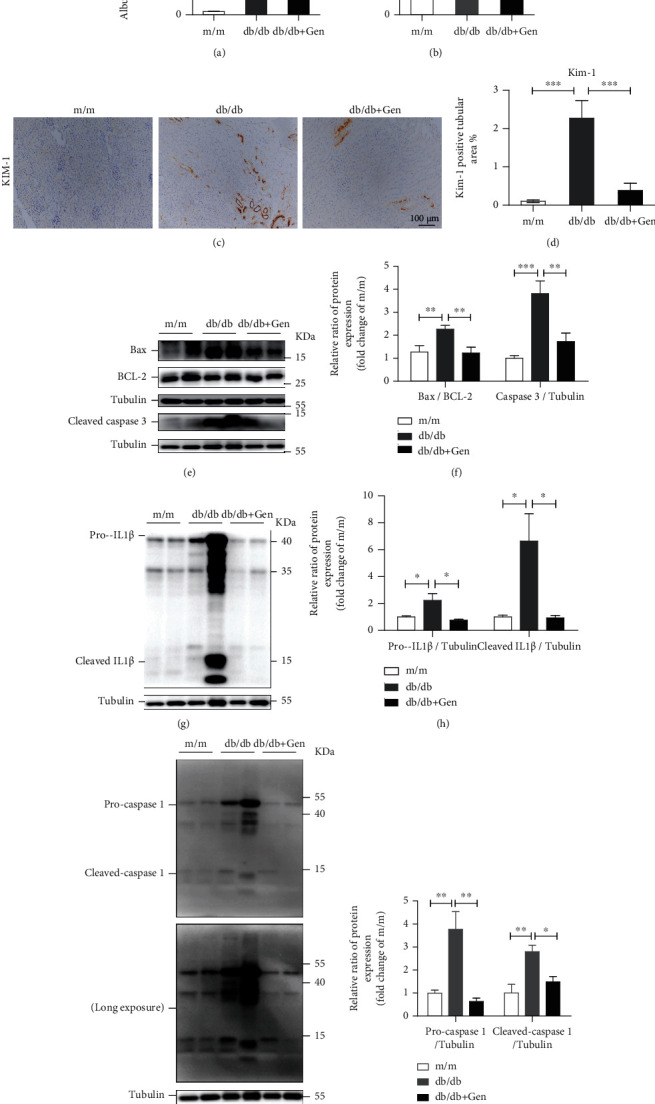
Effect of TFEB activation on renal injury, apoptosis, and inflammation in db/db mice. (a) Albumin-to-creatinine ratio. (b) Serum creatinine level. (c, d) Immunohistochemical staining and quantification of Kim-1 in the renal tissues of mice. Scale bar, 100 *μ*m. (e, f) Western blot assay and quantitative analysis of Bax, BCL-2, and cleaved caspase 3 expression levels in kidneys. (g–j) Western blot assay and quantitative of pro-IL-1*β*, cleaved IL-1*β*, procaspase 1, and cleaved caspase 1 levels in kidneys. Gen: genistein. ^∗^*P* < 0.05, ^∗∗^*P* < 0.01, and ^∗∗∗^*P* < 0.001.

To evaluate the preventive effects of TFEB activation *in vivo*, db/db mice were treated with genistein to induce endogenous TFEB nuclear translocation. We found that db/db mice exhibited an increase microalbuminuria (albumin/creatinine ratio, ACR) and a slight increase in serum creatinine (Scr), while genistein treatment showed significantly reduced levels of ACR with a slight protective effect on Scr (Figures 8(a) and 8(b)). As Kim-1 is a novel biomarker for tubule injury [39], we also found the increased expression of Kim-1 in renal tissues of db/db mice was remarkably alleviated following genistein treatment (Figures 8(c) and 8(d)). Simultaneously, genistein treatment significantly protected cells from apoptosis as judged by the decrease in apoptotic protein cleaved caspase 3 expression and Bax/BCL-2 expression ratio in db/db mouse renal tissues (Figures 8(e) and 8(f)).

It is well known that inflammation plays an essential role in the pathogenesis of DN [40]. Thus, we examined whether genistein treatment invoked an anti-inflammatory effect in db/db mice. As shown in Figures 8(g)–8(j), the increased expression levels of proinflammatory cytokines, including caspase 1 and IL-1*β*, in db/db mice were markedly reduced after genistein treatment, implying an anti-inflammatory effect of TFEB activation by genistein.

Taken together, these results indicate that TFEB activation by genistein alleviated TEC injury under diabetic conditions.

## 4. Discussion

As disruption of lysosomal homeostasis can lead to the tubulopathy of diabetic nephropathy, we evaluated whether decreased activity of TFEB is responsible for disturbed lysosome biogenesis and clearance in this pathological process. The major findings of the present study can be summarized as follows: activated mTOR contributes to TFEB inactivation, therefore perturbing lysosomal homeostasis by impairing lysosomal biogenesis and clearance in TECs of DN. TFEB activation, on the other hand, protects TECs from diabetic injuries by maintaining lysosomal homeostasis.

We have previously shown that pathogenic factors of DN induce LMP and thereby challenging lysosomal homeostasis in TECs [17, 41]. In response to LMP, cellular lysosomal replenishment and lysosomal clearance are enhanced to maintain lysosomal stability under physiological conditions [22, 42, 43]. However, the processes that regulate lysosomal homeostasis in TECs under diabetic conditions remain unclear. In the present study, we observed an accumulation of both damaged lysosomes and autophagy substrates under *in vitro* and *in vivo* diabetic conditions, indicating a severe deficiency in damaged lysosomal clearance. In addition, insufficiency lysosomal replenishment may have been accompanied by deficient lysosomal clearance, as the number of primary lysosomes in diabetic TECs was remarkably reduced. Therefore, it appears that both lysosomal clearance and lysosome replenishment processes were impaired, contributing to the lysosomal homeostasis perturbation in diabetic TECs.

Recent studies have unveiled a crucial role of TFEB in regulation of lysosomal biogenesis and function [25, 44, 45]. It should be noted that the activity of TFEB varies according to different cellular contexts. During starvation, TFEB is activated by dephosphorylation, which enables its translocation to the nucleus [46, 47]. However, under aberrant lysosomal storage conditions, TFEB is inactivated and retained in the cytoplasm [48]. These observations suggest that the subcellular distribution of TFEB is dependent on particular stimuli. In the present study, we found that the insufficient lysosomal replenishment and clearance coincided with TFEB cytoplasm retention and inactivation in human diabetic kidneys and in renal tissues of db/db mice. In addition, the AGE-mediated cytosolic TFEB sequestration contributed to TFEB inactivation notably decreasing the transcription of lysosomal-related genes in diabetic TECs. Thus, our results extend the understanding of the TFEB activity and subcellular distribution under diabetic conditions.

TFEB localizes predominantly in the cytoplasm of resting cells and translocates into nucleus upon dephosphorylation to induce transcription of target genes under conditions of lysosomal stress [49]. Notably, we observed an increase in the levels of phosphorylated TFEB in the kidneys of db/db mice, suggesting that TFEB inactivation may result from the failure of dephosphorylation in DN. Indeed, it has been shown that TFEB is mainly regulated at the posttranslational level via phosphorylation [50], and mTOR has been demonstrated to regulate this process by phosphorylating TFEB at Ser122, Ser 142, and Ser 211 to induce its cytoplasmic retention and inactivation [45, 51, 52]. Consistent with this, we found that the activity of mTOR was dramatically elevated in the kidneys of db/db mice, and that AGE treatment increased the activity of mTOR as well as its lysosomal translocation in cultured TECs. Meanwhile, Torin1, an inhibitor of mTOR, partly reversed AGE-mediated TFEB cytoplasm retention in HK-2 cells, further supporting the critical role of mTOR in the process of TFEB inactivation. However, apart from mTOR, kinases protein kinase C *β* and ERK2 have also been shown to phosphorylate TFEB [49, 53]. Whether these kinases are associated with TFEB inactivation in TECs under diabetic conditions requires further investigation.

Most importantly, our results showed that TFEB overexpression remarkably enhanced the clearance of damaged lysosomes via lysophagy, notably induced biogenesis of new lysosomes to compensate for lysosomal capacity, and increased the degradation capacity of lysosomes in AGE-treated TECs. Recent studies using overexpression of exogenous TFEB have demonstrated the therapeutic effects of TFEB in animal models of storage diseases [54, 55]. However, it is difficult to overexpress TFEB in kidney TECs. Compared with overexpression of TFEB, we found that genistein increased nuclear translocation of TFEB and recovered lysosomal abnormalities both *in vivo* and *in vitro* diabetic TECs. While some may argue that genistein is not a selective TFEB activator [56], the effect of genistein-induced lysosomal biogenesis was almost completely reversed by TFEB silencing in AGE-treated HK-2 cells, indicating that genistein indeed affects lysosomes by activating TFEB. Thus, lysosomal enhancement by both TFEB overexpression and pharmacological activation of endogenous TFEB could potentially be used to maintain lysosomal homeostasis and may be an attractive therapeutic approach to retard the progression of DN.

Emerging evidences have demonstrated that inflammation plays an essential role in the development of DN [57]. Accumulation of AGEs activates proinflammatory mediators such as caspase 1 and IL-1*β* expression [58], which directly induces diabetic renal injury [59]. In our study, TFEB activation by genistein notably reduced the tubule injury. In addition, the attenuated tubule injury in genistein-treated db/db mice was associated with a remarkably inhibited renal inflammation, as evidence by reduced expression of caspase 1 and IL-1*β*. Consistent with our data, TFEB has also been shown to promote an anti-inflammatory phenotype in endothelial cells [60], and it is likely to play a key role in anti-inflammatory activity by enhancing lysosomal activity [61].

In summary, we revealed that the transcriptional activity of TFEB is pivotal for the lysosomal homeostasis in TECs during DN. It is worthy highlighting that upregulation of TFEB was able to restore lysosomal homeostasis in diabetic TECs. Consequently, our findings may be important for developing therapeutic strategies to manipulate lysosomal system through TFEB in DN treatment.

## Figures and Tables

**Table 1:** Clinical characteristics of patients.

Groups	Control	DN	*P* value
Sample size	15	15	-
Male sex (%)^a^	9.0 (60%)	10.0 (66.7%)	0.099
Age (years)^b^	45.2 ± 2.5	52.3 ± 3.5	0.112
Serum glucose (mmol/l)^b^	4.8 ± 0.2	5.9 ± 0.6	0.051
Hemoglobin (g/l)^b^	129.2 ± 3.4	94.0 ± 1.8	<0.001
Serum albumin (g/l)^b^	40.3 ± 1.6	27.7 ± 2.1	<0.001
Total cholesterol (mmol/l)^b^	4.5 ± 0.5	6.2 ± 0.4	0.013
Triglyceride (mmol/l)^b^	1.5 ± 0.3	1.7 ± 0.2	0.722
Blood urea nitrogen (mmol/l)^b^	5.1 ± 0.6	9.7 ± 1.5	0.007
Serum creatinine (*μ*mol/l)^b^	73.5 ± 6.7	202.6 ± 30.8	<0.001
Serum uric acid (*μ*mol/l)^b^	385.8 ± 35.8	361.9 ± 30.2	0.635
24-hour urinary protein (g)^b^	0.2 ± 0.1	5.3 ± 0.7	<0.001
Case number of hypertension (%)^a^	3 (20%)	8 (53.3%)	0.063

^a^Data are presented as counts (%), and the *P* value for the difference between two groups was calculated by the Wilcoxon rank sum test. ^b^Data are presented as mean ± SEM, and the *P* value for the two group comparison was calculated by an independent-sample *t*-test.

## Data Availability

The authors confirm that the data supporting the findings of this study are available within the article and its supplementary materials.

## Supplementary Materials

Supplementary Materials Supplemental Figure S1: autophagy is impaired in diabetic TECs. Supplemental Figure S2: overexpression of TFEB in HK-2 cells. Supplemental Table S1: primer sequences for qRT-PCR.
