# Supplementary material for: Activation of Transcription Factor EB Alleviates Tubular Epithelial Cell Injury via Restoring Lysosomal Homeostasis in Diabetic Nephropathy
Source: Oxid Med Cell Longev. 2022 Jan 12;2022:2812493. doi: 10.1155/2022/2812493 (PMC8786470; doi:10.1155/2022/2812493)
Supplement: Supplementary Materials — Supplemental Figure S1: autophagy is impaired in diabetic TECs. Supplemental Figure S2: overexpression of TFEB in HK-2 cells. Supplemental Table S1: primer sequences for qRT-PCR. [file 2812493.f1.zip › Supplementary information 3.docx]

**Supplementary information**

**Activation of transcription factor EB alleviated tubular epithelial cells injury via restoring lysosomal homeostasis in diabetic nephropathy**

Shujun Wang^1,#^, Kaipeng Jing^1,#^, Hongluan Wu^1^, Xiaoyu Li^1^, Chen Yang^1^, Tingting Li^1^, Haoxuan Tang^1^, Ting Zou^1^, Yao She^1^ and Hua-feng Liu^1 *^

^1^Key Laboratory of Prevention and Management of Chronic Kidney Disease of Zhanjiang City, Institute of Nephrology, Affiliated Hospital of Guangdong Medical University, Zhanjiang, Guangdong 524001, China.

**Supplemental Figure Legends**

***Figure S1*** ***Autophagy is impaired in diabetic TECs.***

(a, b) Representative images of Immunofluorescence staining with anti-LC3 and p62 antibodies in renal cortex from DN patients and db/db mice. (c) Western blot assay and quantitative of LC3 and P62 expression in renal tissues from m/m and db/db mice. (d) Immunofluorescence staining and (e) western blot analysis of LC3 and p62 expression in HK-2 cells exposed to increasing concentrations of AGE-BSA for 12h. Scale bar, 10 μm. **P* ＜0.05, ***P*＜0.01.

***Figure S2 Overexpression of TFEB in HK-2 cells.***

(a) RT-qPCR analyses of TFEB mRNA level in HK-2 cells infected with or without TFEB lentivirus. (b) Western blot assay and (c) quantification of TFEB expression in HK-2 cells infected with or without TFEB lentivirus. ****P*＜0.001.

**Supplemental Table**

**Table S1. Primer sequences for qRT-PCR**

| Gene | Forward sequence (5’-3’) | Reverse sequence (3’-5’) |
| --- | --- | --- |
| TFEB-mouse | AGAGTTGCCCAGTGAGGATG | GGTGATGGAACGGAGACTGT |
| TFEB-human | ACCTGTCCGAGACCTATGGG | CGTCCAGACGCATAATGTTGTC |
| Vps11-human | AGGTCATCAGCGTGTGTGAG | GGTGGCATGAGGTTCTTGTT |
| CLCN7-human | TTCCATGATCTCCACGTTCA | TCGTGTAGGCCATTTTCTCC |
| ATP6V1A-human | GGGTGCAGCCATGTATGAG | TGCGAAGTACAGGATCTCCAA |
| ATP6V1B2-human | AGTCAGTCGGAACTACCTCTC | CTACCGGTAAGGTCAAATGGAC |
| Actin-human | TCTGGCACCACACCTTCTACAATG | AGCACAGCCTGGATAGCAACG |
| Actin-mouse | TGTTACCAACTGGGACGACA | GGGGTGTTGAAGGTCTCAAA |
